# Association between Neonatal Hypoglycemia and 30-Day Breastfeeding Outcomes among Gravidas with Type 2 Diabetes

**DOI:** 10.1055/a-2827-0515

**Published:** 2026-03-30

**Authors:** Daniella Rogerson, Marni Jacobs, Minhazur Sarker, Kim Boggess, Ashley N. Battarbee, Jerrie Refuerzo, Noelia Zork, Gayle Olson, Celeste Durnwald, Kjersti Aagaard, Kedra Wallace, Christina Scifres, Todd Rosen, Sherri Longo, Gladys A. Ramos, on behalf of the MOMPOD Study Consortium

**Affiliations:** 1 Department of Obstetrics, Gynecology, and Reproductive Sciences, University of California San Diego, San Diego, California, United States; 2 Department of Obstetrics & Gynecology, University of North Carolina at Chapel Hill School of Medicine, Chapel Hill, North Carolina, United States; 3 Division of Maternal-Fetal Medicine, University of Alabama at Birmingham Heersink School of Medicine, Birmingham, Alabama, United States; 4 Department of Obstetrics, Gynecology and Reproductive Sciences, University of Texas Health Houston McGovern Medical School, Houston, Texas, United States; 5 Division of Maternal Fetal Medicine, Columbia University Irving Medical Center, New York, New York, United States; 6 Department of Obstetrics and Gynecology, University of Texas Medical Branch, Galveston, Texas, United States; 7 Department of Obstetrics and Gynecology, University of Pennsylvania Perelman School of Medicine, Philadelphia, Pennsylvania, United States; 8 Division of Maternal Fetal Medicine, Baylor College of Medicine and Texas Children's Hospital, Houston, Texas, United States; 9 Department of Obstetrics and Gynecology, University of Mississippi Medical Center, Jackson, Mississippi, United States; 10 Division of Maternal Fetal Medicine, Indiana University School of Medicine, Indianapolis, Indiana, United States; 11 Division of Maternal Fetal Medicine, Rutgers Health/Robert Wood Johnson Medical School, Brunswick, New Jersey, United States; 12 Division of Maternal Fetal Medicine, Ochsner Health, New Orleans, Louisiana, United States

**Keywords:** breastfeeding, type 2 diabetes, neonatal hypoglycemia, MOMPOD

## Abstract

**Objective:**

Pregestational diabetes is associated with low prevalence of breastfeeding due to low rates of intent, delayed lactogenesis, and early infant separation. It is hypothesized that the perceived need for formula supplementation due to neonatal hypoglycemia, coupled with maternal low early milk supply, is a barrier to breastfeeding initiation. Whether there is an association between neonatal hypoglycemia and breastfeeding is unknown. We evaluated associations between neonatal hypoglycemia and breastfeeding.

**Study Design:**

This is a secondary analysis of a randomized controlled trial of metformin versus placebo plus insulin in participants with type 2 diabetes. We included participants who delivered a liveborn neonate, endorsed intention to breastfeed, and had neonatal hypoglycemia data available. A breastfeeding questionnaire was administered at 30-day postpartum, and outcomes were compared between neonates with and without hypoglycemia. The primary outcome was prevalence of exclusive, partial, or no breastfeeding at 30-day postpartum. Secondary outcomes included time to breastfeeding cessation and contributing factors. Characteristics were compared with chi-square, *t* -tests, or Wilcoxon tests.

**Results:**

A total of 420 participants in the primary study (53%) completed an antepartum survey, including a question about intent to breastfeed. After exclusion criteria were applied, 370 (91%) of 405 possible participants reported intention to breastfeed. Among these 370 who met criteria and had intention to breastfeed, 265 (72%) responded to the 30-day postpartum questionnaire. Of these 265, 114 (43%) had neonatal hypoglycemia and 151 (57.0%) did not. Prevalence of not breastfeeding (35 vs. 37%), exclusive breastfeeding (18 vs. 13%), and partial breastfeeding (47 vs. 50%) did not differ between neonates with and without hypoglycemia ( *p* = 0.51). This persisted in a neonatal intensive care unit-admitted subgroup ( *p* = 0.29). Participants who stopped breastfeeding did so on average at 2.6 to 2.8 weeks ( *p* = 0.76).

**Conclusion:**

This study found no impact of neonatal hypoglycemia on 30-day postpartum breastfeeding prevalence among participants with diabetes.

**Key Points:**

- Type 2 diabetes reduces the likelihood of breastfeeding, and the barriers are not fully known.
- Neonatal hypoglycemia is hypothesized to be one barrier to breastfeeding among this population.
- Neonatal hypoglycemia did not impact breastfeeding among diabetic gravidas 30-day postpartum.
- This remained true among neonates admitted to the neonatal intensive care unit.
- Further research examining barriers to breastfeeding among gravidas with diabetes is needed.

## Introduction

Exclusive breastfeeding for the first 6 months of life is recommended by the World Health Organization, the American College of Obstetricians and Gynecologists, and the American Academy of Pediatrics due to significant maternal and neonatal benefits. 1 Pregestational diabetes is associated with decreased exclusive breastfeeding, attributed to lower intentions to breastfeed, delayed lactogenesis, and higher maternal–infant separation and delayed bonding due to cesarean delivery and neonatal intensive care unit (NICU) admission. 1 2

Neonatal hypoglycemia is common among parturients with pregestational diabetes. Hypoglycemia severity, duration, and recurrence determine treatment, which may include feeding, oral dextrose gel, or intravenous dextrose for refractory cases. 3 Neonatal hypoglycemia has been hypothesized to be a barrier to breastfeeding for a number of reasons. 3 4 5 Given the limited volume and lower lactose of early breastmilk, and delayed lactogenesis in persons with diabetes, maternal breastfeeding may be less able to correct hypoglycemia. 6 7 The perception that breastmilk alone cannot treat neonatal hypoglycemia may also lead to early formula feeding, independent of true clinical limitations. These factors together may precipitate early donor breastmilk or formula supplementation in infants with hypoglycemia, interventions that are known barriers to exclusive and long-term breastfeeding. 3 4 5 Early initiation of breastfeeding is a key determinant of long-term breastfeeding outcomes, including among patients with diabetes, and thus even transient neonatal hypoglycemia requiring maternal–infant separation or early feeding with donor breast milk or formula may preclude this early initiation and affect breastfeeding outcomes. 4 8

In recent years, the support for exclusive breastfeeding has increased. 4 It remains poorly understood whether these changes have impacted breastfeeding outcomes among neonates with hypoglycemia born to persons with diabetes. Our objective was to evaluate differences in breastfeeding prevalence at 30-day postpartum among participants with type 2 diabetes with or without neonatal hypoglycemia. We hypothesized that breastfeeding prevalence would be lower among neonates with hypoglycemia.

## Materials and Methods

This is a secondary analysis of a multicenter randomized controlled trial of metformin versus placebo in addition to insulin in participants with singleton, nonanomalous gestations with preexisting type 2 diabetes or newly diagnosed diabetes prior to 22 ^6/7^ weeks. 9

**Fig. 1:**
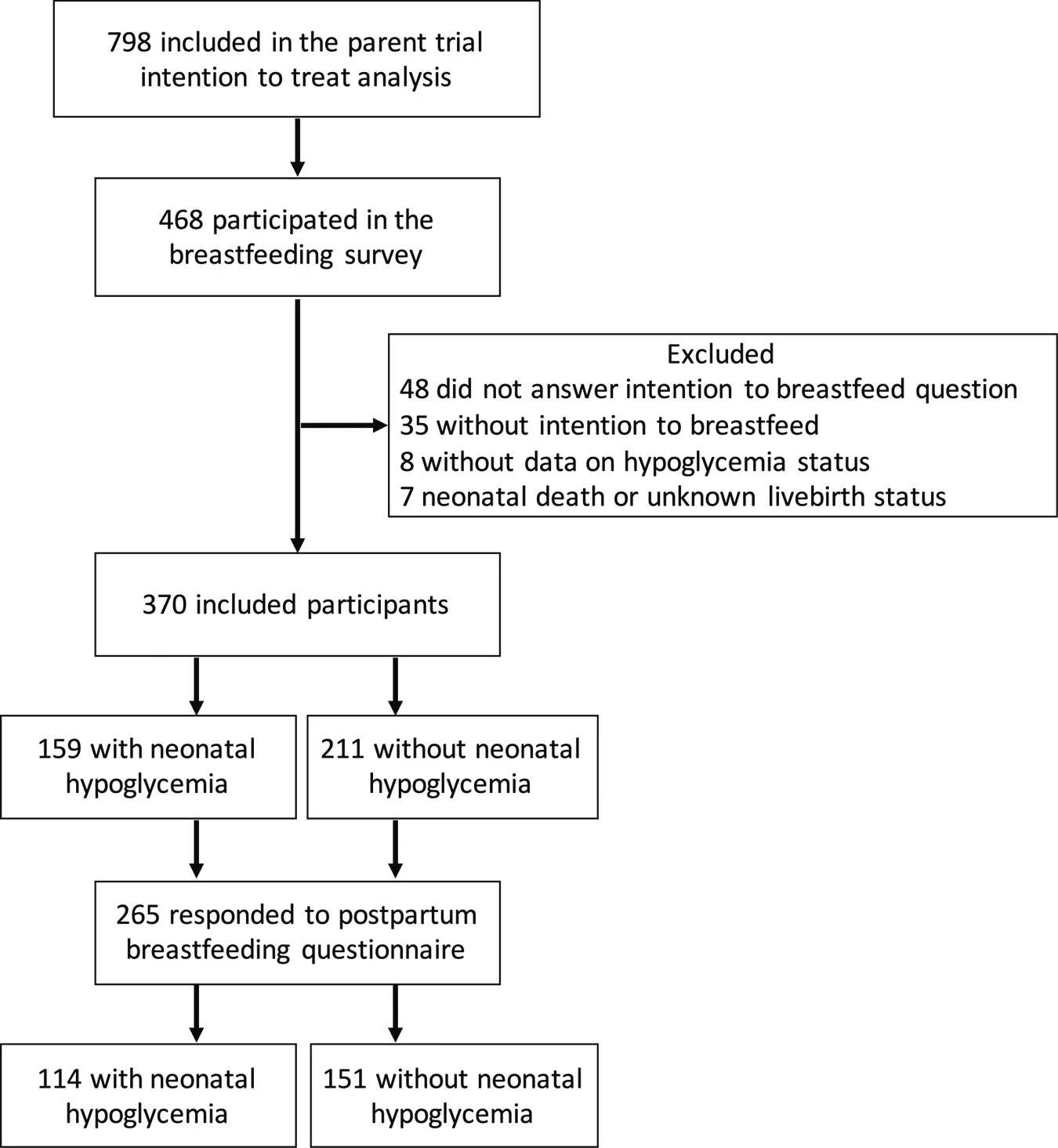
Study flowsheet. Study population after applying inclusion and exclusion criteria.

For this analysis, we included all participants who received at least one dose of study drug, delivered a liveborn neonate, had documented neonatal hypoglycemia metrics, and endorsed intention to breastfeed ( Fig. 1 ). A validated breastfeeding intention survey at 24 to 30 weeks' gestation determined participant feeding intentions along a standardized scale. 10 A follow-up phone survey was administered at 30-day postpartum to determine breastfeeding outcomes (exclusive, partial, or not breastfeeding). 1

We compared 30-day postpartum breastfeeding outcomes among neonates with and without neonatal hypoglycemia. Neonatal hypoglycemia was defined as capillary blood glucose < 40 mg/dL, or requiring intravenous replacement within 72 hours of birth. Treatment of neonatal hypoglycemia was pragmatic and left to the discretion of each provider/center in the parent trial. We performed a subgroup analysis among NICU-admitted neonates with and without neonatal hypoglycemia. Neonates in the hypoglycemia NICU subgroup had at least one admission indication being hypoglycemia.

Primary outcome was the prevalence of breastfeeding at 30-day postpartum categorized as not breastfeeding, exclusive breastfeeding, or partial breastfeeding. Not breastfeeding was defined as having stopped breastfeeding, exclusive breastfeeding was defined as not using formula supplementation within the past week, partial breastfeeding was defined as reported use of both breastmilk and formula supplementation in the past week. Secondary outcomes included time to breastfeeding cessation and factors contributing to breastfeeding cessation.

Categorical variables are reported as frequencies and percentages, and continuous variables are reported as means with standard deviation or median with interquartile ranges, as appropriate. Baseline characteristics and outcomes were compared with chi-square, *t* -tests, or Wilcoxon tests. A *p* -value less than 0.05 was considered statistically significant. Statistical analyses were performed on SAS 9.4.

The parent trial protocol was approved by the institutional review board of each clinical site, and the trial was registered on ClinicalTrials.gov with Identifier NCT02932475. All patients provided voluntary written informed consent prior to participation. This secondary analysis did not require further oversight from the institutional review board, since deidentified data were used.

## Results

Of 796 participants in the parent trial, 420 (53%) completed the antepartum breastfeeding survey and answered the intent to breastfeed question. After exclusions, there were 405 eligible participants; of them, 370 (91%) reported intention to breastfeed, whereas 35 of 405 (7%) endorsed intention not to breastfeed and were also excluded. Among the 370 included participants, 265 (72%) completed the postpartum survey, and 71% of surveys were completed between 20 and 40 days' postpartum. Participants who completed the postpartum survey were more likely to be Hispanic (57 vs. 37%, *p* = 0.003) than those who did not; there were no other significant demographic differences (data not shown). Of those who completed the postpartum survey, 114 (43.0%) experienced neonatal hypoglycemia and 151 (57.0%) did not ( Fig. 1 ). Baseline demographics are presented in Table 1 . Those with neonatal hypoglycemia were born at earlier gestation (36.5 vs. 37.6 weeks, *p* < 0.0001), more likely to deliver via cesarean (73 vs. 58% cesarean, *p* = 0.04), have received antenatal corticosteroids (81% no steroids vs. 93%, *p* = 0.001), and be admitted to the NICU (64 vs. 19%, *p* < 0.0001) with longer hospital stay (4 vs. 2 days, *p* < 0.0001). There were no differences between groups in maternal age, maternal body mass index, birthweight, ethnicity, level of education, insurance type, parity, or metformin use.

**Table 1:** Baseline demographics for neonates with and without neonatal hypoglycemia among participants intending to breastfeed

Demographic	Neonatal hypoglycemia ( *n* = 159, 43.0%)	No neonatal hypoglycemia ( *n* = 211, 57.0%)	*p* -Value
Maternal age (y), mean (SD)	32.7 (6.0)	33.5 (5.2)	0.17
Maternal BMI (kg/m ^2^ ), mean (SD)	36.6 (8.1)	36.8 (8.8)	0.85
Hispanic ethnicity, *n* (%)	77 (48.4)	113 (53.6)	0.33
Highest level of education, *n* (%) Less than high school High school diploma/GED College degree or higher	31 (20.3) 71 (46.4) 51 (33.3)	44 (22.1) 96 (48.2) 59 (29.7)	0.75
Insurance, *n* (%) Private/Tricare Public No insurance	29 (18.2) 123 (77.4) 7 (4.4)	46 (21.9) 149 (71.0) 15 (7.1)	0.33
Parity, median (IQR)	2 (1, 3)	2 (1, 3)	0.68
Mode of delivery, *n* (%) Vaginal Cesarean	43 (27.0) 116 (73.0)	88 (41.7) 123 (58.3)	0.004
GA at delivery (wk), mean (SD)	36.5 (2.3)	37.6 (1.9)	<0.0001
Preterm birth, *n* (%)	64 (40.3)	46 (21.8)	0.0001
Birthweight, mean, g (SD)	3166 (826)	3191 (647)	0.75
Neonatal LOS, median, d (IQR)	4 (2, 10)	2 (2, 3)	<0.0001
NICU admission, *n* (%)	102 (64.1)	40 (19.0)	<0.0001
NICU LOS, median, d (IQR)	8 (4, 24)	6 (2, 15.5)	0.29
Steroids for fetal lung maturity, *n* (%) None Partial course Full course	129 (81.1) 7 (4.4) 23 (14.5)	196 (92.9) 1 (0.5) 14 (6.6)	0.001
Treatment group, *n* (%) Metformin Placebo	74 (46.5) 85 (53.5)	117 (55.5) 94 (44.5)	0.09

Abbreviations: BMI, body mass index; GA, gestational age; IQR, interquartile range; LOS, length of stay; NICU, neonatal intensive care unit; SD, standard deviation.

**Fig. 2:**
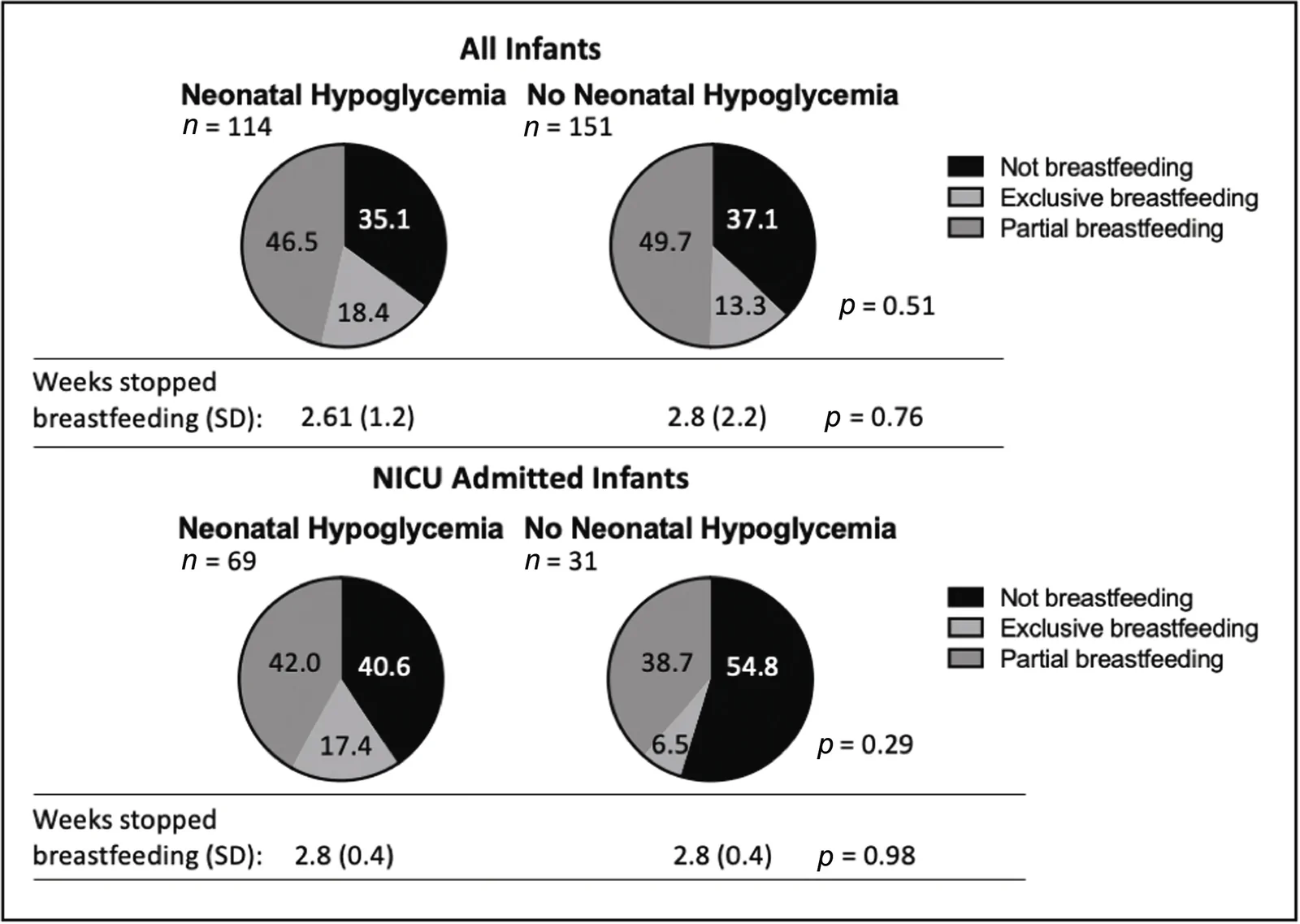
Breastfeeding outcomes compared by neonates with and without neonatal hypoglycemia among all participants intending to breastfeed and the subgroup of participants requiring neonatal intensive care unit admission.

Among 265 participants who completed the postpartum questionnaire, the prevalence of not breastfeeding, exclusive breastfeeding, and partial breastfeeding were 36% ( *n* = 96), 16% ( *n* = 41), and 48% ( *n* = 128), respectively. The majority of participants (64%, 169/265) reported some form of breastfeeding at 30-day postpartum. Prevalence of not breastfeeding (35 vs. 37%), exclusive breastfeeding (18 vs. 13%), and partial breastfeeding (47 vs. 50%) did not significantly differ between neonates with and without neonatal hypoglycemia, respectively ( *p* = 0.51, Fig. 2 ). This remained true in a subanalysis of NICU-admitted neonates ( *p* = 0.29). Participants who stopped breastfeeding did so at an average of 2.6 ± 1.2 weeks' postpartum in the hypoglycemia group and 2.8 ± 2.2 weeks in the no hypoglycemia group ( *p* = 0.76, Fig. 2 ). Participants who stopped breastfeeding reported doing so for similar reasons in both groups, including trouble latching, maternal pain, poor neonatal weight gain or satisfaction, low milk supply, and ability to leave the newborn for several hours ( Table 2 ). Though not significant, breastmilk not satisfying the infant, poor neonatal weight gain, and not enough milk were more frequently cited as reasons for breastfeeding cessation among the neonatal hypoglycemia group ( Table 2 ). Among the 35 participants without initial breastfeeding intention, 7 reported breastfeeding on the postpartum survey.

**Table 2:** Indications for breastfeeding cessation among participants who stopped breastfeeding by 30-day postpartum

Reasons for cessation, *n* (%)	Neonatal hypoglycemia ( *n* = 33)	No neonatal hypoglycemia ( *n* = 66)	*p* -Value
Trouble sucking/latching Not at all important Not very important Somewhat important Very important	12 (36.4) 3 (9.1) 5 (15.2) 13 (39.4)	22 (33.3) 3 (4.6) 9 (13.6) 32 (48.5)	0.69
Breast milk did not satisfy Not at all important Not very important Somewhat important Very important	7 (21.2) 1 (3.0) 6 (18.2) 19 (57.6)	24 (36.9) 6 (9.2) 10 (15.4) 25 (38.5)	0.21
Baby not gaining weight Not at all important Not very important Somewhat important Very important	13 (39.4) 4 (12.1) 3 (9.1) 13 (39.4)	31 (47.7) 8 (12.3) 9 (13.8) 17 (26.2)	0.62
Didn't have enough milk Not at all important Not very important Somewhat important Very important	6 (18.7) 2 (6.3) 6 (18.8) 18 (56.2)	18 (27.3) 3 (4.5) 7 (10.6) 38 (57.6)	0.61
Sore nipples Not at all important Not very important Somewhat important Very important	17 (53.1) 8 (25.0) 3 (9.4) 4 (12.5)	40 (61.5) 10 (15.4) 6 (9.2) 9 (13.9)	0.69
Too painful Not at all important Not very important Somewhat important Very important	19 (57.6) 7 (21.2) 2 (6.1) 5 (15.1)	37 (56.9) 15 (23.1) 3 (4.6) 10 (15.4)	1.00
Wanted to be able to leave Not at all important Not very important Somewhat important Very important	22 (68.8) 6 (18.7) 1 (3.1) 3 (9.4)	40 (61.5) 7 (10.8) 7 (10.8) 11 (16.9)	0.34

## Discussion

Among participants with type 2 diabetes, prevalence of exclusive and partial breastfeeding at 30-day postpartum did not differ by presence or absence of neonatal hypoglycemia. This remained true in a subanalysis of neonates with NICU admission. Participants who stopped breastfeeding did so at similar times postpartum, regardless of neonatal hypoglycemia status. Among individuals that stopped breastfeeding, similar reasons were reported as contributing factors.

The prevalence of any breastfeeding at 30-day postpartum in this cohort of participants with type 2 diabetes was 64%, higher than reported in other studies, but lower than the general population. 2 Our findings at 30-day postpartum are consistent with the retrospective analysis by Cordero et al. showing no effect of neonatal hypoglycemia on breastfeeding initiation at time of discharge for neonates born to participants with diabetes, with and without NICU admission. 11 Thus, our findings indicate that with contemporary breastfeeding practices, neonatal hypoglycemia does not appear to pose a significant additional barrier among people who intended to breastfeed. As our study only included participants who intended to breastfeed, it cannot comment on the impact of neonatal hypoglycemia among participants who were undecided on method of feeding. Given reported lower intention to breastfeed among persons with diabetes, 2 a cohort of undecided participants could be examined to identify modifiable factors, which influences final method of feeding. Our understanding of factors that influence intention to breastfeed among this population is also limited, but given the clear and evident benefits for restoring maternal metabolic physiology, should be a priority for research.

While characterizing barriers to breastfeeding cessation is important, it is equally as important to understand protective factors enabling breastfeeding among persons with pregestational diabetes. For example, the number of attempts at breastfeeding in the NICU has been positively correlated with breastfeeding outcomes. 12 Additionally, early breastfeeding among newborns of people with diabetes at risk for neonatal hypoglycemia has been shown to correlate with ongoing breastfeeding at discharge, compared with early formula feeding, regardless of NICU admission. 4 Both of these highlight how early lactational support among persons with preexisting diabetes may improve sustained breastfeeding, and interventions like these may explain similar breastfeeding outcomes in neonates with and without hypoglycemia.

Our study has limitations. Our cohort may be underpowered to detect small differences in breastfeeding outcomes. The parent trial was conducted across large medical centers where attitudes toward breastfeeding and lactation support may not be generalizable to community settings. Participants were not specifically asked whether providers suggested the use of formula or donor milk in response to neonatal hypoglycemia or otherwise, or about specific barriers to early initiation of breastfeeding in the hospital when neonatal hypoglycemia is most likely to be pertinent. Multiple reasons for breastfeeding cessation were reported without order of significance, precluding assessment of possible interventions for the most significant barriers. Participants with type 1 diabetes were not included. Quality metrics to assess breastfeeding prevalence and NICU lactation support likely differed between sites, although these factors make study findings more generalizable. Our study also has strengths. Response to breastfeeding questionnaires 30-day postpartum was high (72%), which allowed detailed outcomes to be captured. The geographically diverse nature of the cohort further increases generalizability. Although there are numerous possible confounders for breastfeeding, including known differences in breastfeeding among patients of different cultural backgrounds and education levels, 13 no significant differences were seen between the group of infants with and without hypoglycemia in terms of age, parity, ethnicity, insurance, and education level.

## Conclusion

Our secondary analysis found no impact of neonatal hypoglycemia on breastfeeding at 30-day postpartum among neonates of participants with diabetes. Given the benefits of breastfeeding, further studies are needed to identify interventions that increase sustained breastfeeding among this population.
